# Control of *Listeria monocytogenes* on Frankfurters by Surface Treatment with Olive Mill Wastewater Polyphenolic Extract

**DOI:** 10.3390/foods14050774

**Published:** 2025-02-24

**Authors:** Rossana Roila, Andrea Valiani, Maurizio Servili, David Ranucci, Roberta Galarini, Roberta Ortenzi, Sara Primavilla, Raffaella Branciari

**Affiliations:** 1 Department of Veterinary Medicine, University of Perugia, Via San Costanzo 4, 06121 Perugia, Italy; rossana.roila@unipg.it (R.R.); raffaella.branciari@unipg.it (R.B.); 2 Istituto Zooprofilattico Sperimentale dell’ Umbria e delle Marche “Togo Rosati”, Via Salvemini 1, 06126 Perugia, Italy; r.galarini@izsum.it (R.G.); r.ortenzi@izsum.it (R.O.); s.primavilla@izsum.it (S.P.); 3 Department of Agricultural, Food and Environmental Sciences, University of Perugia, Via S. Costanzo, 06126 Perugia, Italy; maurizio.servili@unipg.it

**Keywords:** bioactive compounds, color stability, food safety, hydroxytyrosol, sensory properties, shelf life, tyrosol

## Abstract

*Listeria monocytogenes* (LM) is a frequent post-process contaminant in meat products. This study aimed to investigate the antilisterial effectiveness of post-process antimicrobial treatments employing olive mill wastewater polyphenolic extract (PE) in commercially manufactured frankfurters. Frankfurters were inoculated on the surface with a three-strain LM mixture (~10^2^ CFU/g), treated on the surface with PE in a 2-fold series of concentrations (PM, 2PM, 4PM) and a control group (CTR) of PE-untreated samples. Then, the frankfurters were vacuum-packed and stored at 4 °C for 28 days. Samples were examined on days 0, 7, 14, 21, and 28 for LM count, in silico growth modeling, and impacts on pH, water activity (a_w_), and sensory characteristics. From T(time) 7, the PE treatment showed a significant effect on LM growth, registering maximum Δ values between CTR and 4PM of 3.19, 4.86, 4.59, 4.39 at T7, T14, T21, T28, respectively. Minimum effect was attributable to PM treatment with Δ values (CTR versus PM) of 2.07, 2.52, 1.14, 0.65 at T7, T14, T21, T28, respectively. No significant changes occurred in pH (average 6 at T0 and 5.9 at T28), a_w_ (average 0.978 at T0 and 0.968 at T28), nor in sensory profile (*p* > 0.05). These findings suggest that PE is an effective natural antimicrobial, offering a promising approach to enhancing food safety and extending shelf life in meat products.

## 1. Introduction

Listeriosis is a highly prevalent zoonotic disease that is recognized globally for its severity and significance as a foodborne illness and therefore it is rigorously monitored on a global scale [1,2,3]. In 2023, 27 Member States reported 2952 confirmed invasive human *Listeria monocytogenes* cases, resulting in an EU notification rate of 0.66 cases per 100,000 people. This represented a 5.8% rise over the average in 2022 (0.63 instances per 100,000 population), and the highest rate and number of cases reported since 2007 [3].

**Figure 1:**
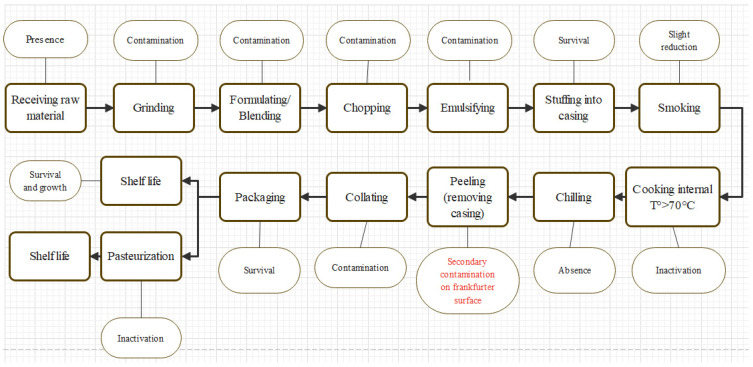
A flowchart depicting the typical process involved in frankfurter production, including the behavior and interaction of *Listeria monocytogenes* at each stage of the operation (oval shapes represent the behavior of *Listeria monocytogenes* and rectangular shapes indicate process steps); references: [7,8].

Listeriosis outbreaks have been primarily linked to the consumption of contaminated ready-to-eat (RTE) foods prepared with red meats, poultry, fish, and seafood, which have been shown to act as LM vehicles [3,4,5,6]. The presence of LM in RTE meat products is mainly attributed to mishandling after thermal treatment, as the bacteria are typically inactivated during this process. For instance, in the production of meat products, such as frankfurters, the critical steps are represented by peeling the casings before vacuum-packaging, packaging, and decontamination after packaging (Figure 1).

Indeed, frankfurters are typically contaminated on the surface after the cooking process before packaging [9]. Once attached to the surface of food products, microorganisms appear to be more difficult to remove [10] and are able to proliferate as neither vacuum-sealed packaging nor refrigeration temperatures are able to inhibit its growth [11,12,13]. Post-process interventions, either alone or in combination with antimicrobial compounds may be used to inactivate and prevent the proliferation of LM on the surface product [9]. Furthermore, despite producers’ recommendations, these products are often consumed without being cooked and are frequently subjected to temperature abuse, making them an ideal medium for the growing of LM [2,13]. Consumption of commercially prepared frankfurters that were contaminated during post-processing operations has been connected to multiple listeriosis outbreaks [3,14]. Implementing post-lethality interventions in meat-processing facilities represents a suitable approach to control LM; among these, the use of natural antimicrobial agents able to limit LM growth during refrigerated storage could be a valuable strategy with several advantages [12]. Indeed, in light of rising consumers’ awareness concerning nutritional and sustainability issues, food manufacturers are seeking for natural and green alternatives to achieve microbiological stability [15]. Various spices, essential oils, plant, and plant by-product extracts used in the meat production process have been investigated in the past few years to evaluate their efficacy against pathogenic bacteria in food products [2,11,16]. Research studies have identified numerous active compounds found in natural extracts that demonstrate remarkable antibacterial and antioxidant properties [17]. Olive oil by-products extracts are well known for their high concentration of hydrophilic phenols, particularly secoiridoids, characteristic of the Oleaceae family, which have demonstrated the ability to inhibit or delay the growth of various Gram-positive and Gram-negative bacteria, both pathogenic and non-pathogenic, in addition to exhibiting antioxidant properties [17,18,19,20]. However, there are no reports on their antimicrobial efficacy when used as an antimicrobial treatment on frankfurters. This study aims to answer the following research questions and formulate hypotheses based on the background information provided.

Research questions: What is the effective dosage of olive oil by-product polyphenol extract (PE) necessary to inactivate *Listeria monocytogenes* (LM) in vitro? How does PE surface treatment affect the growth potential of LM in frankfurters? What are the impacts of PE on the physical attributes and sensory properties of frankfurters?

Hypothesis: Polyphenols obtained from olive oil by-products demonstrate a significant antibacterial effect against LM in vitro, effectively reducing or inhibiting its growth at a specific concentration. The application of polyphenol extract on frankfurter surfaces will reduce the growth potential of LM in comparison to untreated samples. The addition of PE will not adversely affect the sensory properties and physical characteristics (pH, a_w_, color) of frankfurters.

## 2. Materials and Methods

### 2.1. Polyphenolic Extract from Olive Mill Processing By-Products

The polyphenolic extract was obtained by treating olive mill wastewater (*Olea europaea* L. cv. “Koroneiki”) with hydrolytic enzymes, subsequently filtered by means of a membrane process, and encapsulated with a food-grade maltodextrin carrier (1:1 dw), followed by lyophilization (freeze-drying technique at −55 °C, 0.1 mbar) and a grinding process. The extract is registered as food-grade (General Chemical State Laboratory of Greece, certificate n. 30/003/000/3810).

Polyphenolic characterization of the extracts was performed by liquid chromatography quadrupole time-of-flight spectrometry (LC-QTOF-LC-TripleTOF 6600, Sciex, Framingham, MA, USA). Sample extraction, phenol identification, and instrumental conditions were the same as described by Roila et al. [19]. The specific content of polyphenol extract was 14.7 ± 0.3 mg/g hydroxytyrosol, 4.0 ± 0.2 mg/g tyrosol, and 0.3 ± 0.1 mg/g vanillic. The sum of polyphenols found was 19.0 mg/g.

### 2.2. Preliminary In Vitro Antimicrobial Activity Assay

The in vitro evaluation of the antimicrobial activity of PE was conducted to determine the appropriate concentrations to use for the in vivo study, and was conducted by the broth microdilution method defining the minimal inhibitory concentration (MIC) and the minimal bactericidal concentration (MBC), following the instruction of the Clinical Laboratory Standards Institute (CLSI) guidelines [21]. The LM strains tested for the assay were authenticated reference strain WDCM 00021 and two strains from the Istituto Zooprofilattico Sperimentale dell’Umbria and Marche “Togo Rosati” IZSUM collection (ST155, CC155, serotype 1/2a, pork product isolate and ST1, CC1 serotype 4b, pork sausage isolate). The two strains were selected due to their isolation from pork meat products, indicating their adaption to the product environment of this study; each strain was kept at −80 °C in the IZSUM collection. Each bacterial culture was regenerated into brain heart infusion broth (BHI; Oxoid, Basingstoke, UK) by incubation at 24 h at 37 °C. For each microorganism, colonies were resuspended in sterile saline solution and suspension turbidity was measured spectrophotometrically at an optical density of 600 nm (OD600). The microorganism’s suspension to be utilized for the trial was obtained by adjusting the concentration to 5 × 10^5^ CFU/mL, in fresh Mueller Hinton broth (Biolife Italiana s.r.l., Milan, IT) and by vigorous vortexing. An aliquot of each suspension (100 μL) was added to a 96-well microplate containing the same volumes of two-fold serial dilution of the extracts, ranging from 0.5 to 0.0039 g PE/mL. Two controls were added to the assay: a positive control using antibiotic control culture medium with tetracycline (Sigma-Aldrich, St. Louis, MO, USA), organism control (culture medium and bacterial suspension), and negative control (culture broth and PE solution). The plates were incubated for 24 h at 37 °C. MIC was defined, visually, as the lowest concentration of extract that produced no bacterial growth. The MBC was determined by subculturing 10 μL of microwells corresponding to MIC and plated onto fresh 5% sheep blood agar dishes (Microbiol s.r.l., Cagliari, Italy) and then incubated for 24 h at 37 °C. The MBC was represented as the lowest PE concentration capable of killing LM. All tests were performed in triplicate.

### 2.3. Evaluation of PE Antimicrobial Activity on Frankfurters

#### 2.3.1. Experimental Contamination and PE Treatment

The inoculum for the contamination of frankfurter was prepared according to the guidelines of the European Union Reference Laboratory for *Listeria monocytogenes* (EURL Lm, 2021) [22]. A multi-strain mix of the three abovementioned LM strains was used. As for the in vitro assay, frozen stocks of bacterial cultures were regenerated and, subsequently, aliquots of each revitalized culture (100 μL) were transferred into tubes containing BHI (Oxoid, Basingstoke, UK) and incubated for 96 h at 8 ± 1 °C. At the end of the acclimatization time, the mixed culture was serially diluted with saline solution to reach an inoculum concentration of 10^4^ CFU/mL to experimentally contaminate frankfurters. Commercial packages of pork meat frankfurters were purchased from a local supermarket (Perugia, central Italy). The “use by” date was 28 days later than the date of purchase. Each frankfurter was 3 cm in length with a total surface of 25 cm^2^ and a weight of 10 g. Frankfurter was placed in a sterile bag and contaminated with five spots of 20 µL (0.1 mL total) of the bacterial inoculum to reach a final LM concentration of approximately 10^2^ CFU per g of product. The frankfurters were massaged to ensure uniform distribution of the inoculum across the product surface. Following inoculation, the samples were stored at 4 °C for 30 min to facilitate bacterial attachment. This study evaluated three antimicrobial interventions applied to previously LM-contaminated frankfurters that were removed from their inoculation bags and divided into three groups, as follows: PM (polyphenols mix) group, samples treated with 0.1 mL of PE solution at a concentration of 29.5 mg/mL total polyphenols; 2PM group, samples treated with 0.1 mL of PE solution at a concentration of 59 mg/mL total polyphenols; 4PM group, samples treated with 0.1 mL of PE solution at a concentration of 118 mg/mL total polyphenols. Frankfurters from each treatment were removed from the bags in which they were inoculated and vacuum packaged in new vacuum bags (one frankfurter per bag). The concentration used for the experimental polyphenolic treatment was represented by a 2-fold series of concentrations of the bioactive value recorded in the in vitro experiment, aiming to account for the complexity of the food matrix and possible inhibitory/competitive factors as already reported by other authors [8]. For the control group (CTR), LM-contaminated samples were treated with 0.1 mL of sterile distilled water instead of PE solution. The uniform application of the PE solution on the frankfurter surface was performed as previously described for LM contamination. Following the PE treatments (considered Day 0), the frankfurters were vacuum packaged and stored at 4 °C for 28 days in a professional display refrigerator equipped with an internal probe and alarm system set at a temperature variation of 0.5 °C (Angelantoni Life Science, Perugia, Italy). To ascertain whether the experimental treatment affects the physicochemical and sensory characteristics of frankfurters, equivalent experimental groups (PM, 2PM, 4PM, CTR) were produced without LM. The trial session was performed in triplicate. All analyses were carried out on three replicates for each trial session (N = 3 batch; n = 3 replicates per sampling time).

#### 2.3.2. Determination of LM

Before the experimental contamination, the frankfurter batch used in the trial was tested to assess the absence of LM according to ISO 11290-1:2017 [23]. The LM enumeration on samples belonging to the four experimental groups was determined on days 0, 7, 14, 21, and 28 of 4 °C storage following the ISO 11290-2:2017 reference method [24]. All analyses were carried out on three replicates for each trial session (N = 3 batch; n = 3 replicates per sampling time).

#### 2.3.3. In Silico Evaluation of Growth Dynamics

The growth curves for all experimental groups (CTR, PM, 2PM, and 4PM) were defined using DMFit tool (online freeware ComBase database) by fitting the experimental LM enumeration data to the model of Baranyi and Roberts [25]. This method enabled the definition of the kinetic parameters including initial values (Log CFU/g), duration of the lag phase (λ, hours), maximum growth rate (μmax, Log CFU/g/h), and final values (Log CFU/g).

### 2.4. Evaluation of PE Effect on Quality Characteristics of Uncontaminated Frankfurters

#### 2.4.1. Physicochemical Determinations

The pH measurements were performed on days 0, 7, 14, 21, and 28 by placing a pH probe (Mettler Toledo Inc., Columbus, OH, USA) into homogenized (Ultra-Turrax T 25 BASIC, Ika-Werke, Staufen, Germany) samples from the CTR, PM, 2PM, and 4PM formulations that were prepared by first blending the frankfurters and subsequently mixed with deionized water in a 1:9 ratio. Calibration of the pH meter was conducted using phosphate buffers of pH 4.1, 7.1, and 9.6. For each sample, duplicate readings were taken.

The color measurements were performed on the frankfurter surface at 0 and 28 days by determining color coordinates (CIE, 1976) using a Minolta Chromameter CR400 (Minolta, Osaka, Japan—light source of D65 calibrated against a standard white tile) [26]. The results are presented as lightness (L*), redness (a*), and yellowness (b*). To investigate variations in color values and to verify potentially visually perceived differences, the calculation of ΔE was conducted using the following equation: ΔE = [(L* − L*_0_)^2^ + (a* − a*_0_)^2^ + (b* − b*_0_)^2^]^0.5^; where ΔE is defined as the square root of the sum of squares of the differences between L*, a*, and b* coordinates of the treated sample and control.

#### 2.4.2. Sensory Evaluation of Frankfurters

In order to ascertain a perceptible sensory difference among PE-treated frankfurters, triangle tests based on ISO 4120:2007 [27] were performed with 30 untrained assessors who had experience and familiarity with the product. In brief, the taste of frankfurters containing different amounts of PE (PM, 2PM, and 4PM) was compared to the CTR group. Each assessor had to identify the treated sample compared with CTR samples (with α = 0.05; β = 0.05). A preference test utilizing a 12-trained member panel consisting of 7 females and 5 males with higher levels of education (Master’s degree or higher), aged 20 to 60, in accordance with ISO 8587:2006 was also performed at T0 and at T28 [28]. The samples were ranked from 1 (“the least preferred”) to 4 (“the most preferred”). Additionally, the assessors were asked to justify their choices.

### 2.5. Statistical Analysis

Data were analyzed using the GLM procedure (*JMP^®^ 9.0*, SAS Institute Inc., Cary, NC, USA, 2010) [29]. An ANOVA model was used with sample (CTR, PM, 2PM, 4PM) and time (0, 7, 14, 21, 28) as fixed factors. The replicate effect was determined to be non-significant and was consequently eliminated from the model. Tukey’s test was employed to identify the differences in the means, which were deemed significant when *p* < 0.05. The DMFit tool of the free predictive microbiology software ComBase (https://combase.errc.ars.usda.gov; accessed on 20 October 2024) was used to assess the impact of formulation on the growth of the target microorganism. This tool enables the definition of growth parameters, including the lag phase duration and maximum growth rate, using the Baranyi and Roberts model [25]. The fitted results of DMFit were analyzed by the ANOVA model with sample (CTR, PM, 2PM, 4PM) and time (0, 7, 14, 21, 28) as fixed factors. To ascertain a perceptible sensory difference among the samples, triangle tests were performed following ISO 4120:2007 [27], in which assessors were assumed to be able to discriminate with 50% accuracy. To achieve statistical significance at a level of 0.05 for both α-risk (probability of concluding that a perceptible difference exists when one does not) and β-risk (probability of concluding that no perceptible difference exists when one does), 30 assessors were used. For statistical analysis, the results of the discrimination tests were applied to the significance tables in ISO 4120:2007 [27]. The results of the ranking preference test were analyzed by non-parametric Friedman’s test (*p* < 0.01) (ISO 8587:2006), comparing the result obtained with a value of the chi-square parameter (χ^2^) and calculated as a function of the number of samples and the number of consumers [28].

## 3. Results and Discussions

The preliminary evaluation of the PE’s antimicrobial activity against the three strains (reference and izs collection strains) of LM revealed a MIC and MBC value of 15.6 mg/mL. Albeit a comparison of the outcomes in the literature is difficult primarily due to the different composition of polyphenolic extracts, similar effects on LM are reported [17]. Guo et al. [30] tested olive oil polyphenolic extract on LM and showed that the bacterial colony did not grow at a concentration of 1.25 mg/mL. The authors assert that olive oil polyphenolic extract influenced intracellular ATP concentration and cell membrane potential, resulting in decreased bacterial protein and DNA levels, along with alterations in cell morphology. Liu et al. [31] investigated the antimicrobial activity of olive leaf extract on LM and reported that, at a concentration of 62.5 mg/mL, the growth of the pathogen was completely inhibited due to cell membrane damage and loss of flagella. Other in vitro research examining the antibacterial activities of *Olea europaea* polyphenols extracts and their principal constituent, hydroxytyrosol, proved their efficacy against bacteria [32]. The current theory regarding the mechanism of action of olive oil polyphenol extract is to act by destabilizing the microbial cell surface and cytoplasmic membranes [33]. This may result in irreversible damage to the cell membrane and internal structures, the coagulation of cellular compartments, and the inhibition of intracellular enzymes [34]. A research study demonstrated that *B. cereus* exposed to olive polyphenol extract exhibited reduced intracellular ATP levels and bacterial protein content, depolarized cell membranes, and impaired retention of intracellular components [35,36]. Amini et al. [36] conducted a detailed investigation on the impact of *Olea europaea* polyphenols on the intracellular ATP content of bacteria, specifically focusing on their relationship with ATP synthase, an enzyme that plays a direct role in generating energy in the form of ATP, in *E. coli*. Upon binding to the polyphenol binding pocket of ATP synthase, the authors noted a decrease in the enzyme’s activity [36]. This reduction in activity has a direct impact on microbial metabolism, ultimately resulting in the death of the microorganisms. In vitro screening is the first step to define the antimicrobial activity of polyphenolic extracts; however, in situ research is necessary to deepen this effect on food systems that could be modified by the interaction between phenolic compounds and food components, such as lipids and proteins [37]. The results of LM growth in contaminated frankfurters treated with different concentrations of PE are presented in Table 1. The pathogen was not detected in the negative control samples.

There was significant interaction between treatment and time of storage (days 0 to 28) (*p* < 0.001), suggesting that the treatment concentration and time of storage affect the Log number, and the effect of treatment also depends on the storage time. It was observed that the initial population of LM (about 2 Log CFU/g) in CTR samples increased to 5.5 Log CFU/g after 7 days to reach a final value of 8.39 Log CFU/g after 28 days of refrigerated storage. The PM and 2PM treatments were effective in inhibiting the pathogen compared to the CTR. Indeed, after 7 days of storage the LM population was lower by 2 Logs in the two treated groups compared to CTR, while at 14 days of storage, the LM growth was approximately 3 Log CFU/g lower. The most effective treatment was 4PM, which limited the growth of the LM population, reaching approximately 4 Log CFU/g even after 28 days of storage at 4 °C, half the population of CTR.

**Figure 2:**
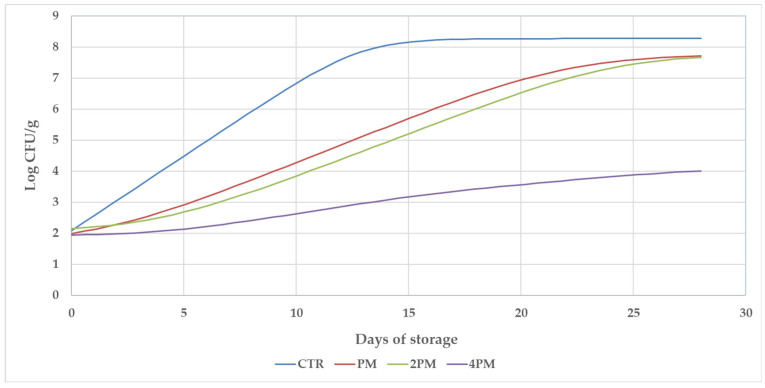
Growth profile of *Listeria monocytogenes* (Log CFU/g) during pork frankfurter storage. Data represent the best fitting of experimental growth-data average values ± standard deviation analyzed with ComBase DMFit software Tool. CTR = control group; PM = frankfurter treated with 29.5 mg/mL of total polyphenols; 2PM = frankfurter treated with 59 mg/mL of total polyphenols; 4PM = frankfurter treated with 118 mg/mL of total polyphenols.

The growth curves of artificially inoculated LM on pork frankfurters with different concentrations of PE are depicted in Figure 2. The respective growth kinetics parameters are reported in Table 2.

The initial values of approximately 10^2^ CFU/g are statistically equivalent for all experimental groups (*p* > 0.05). As expected, the concentration of LM in CTR increased steadily in the first days of shelf-life (Figure 2) as also confirmed by the highest values (*p* < 0.001) of the maximum growth rate (µmax) that defines the slope of the curve in the exponential growth phase. Furthermore, the CTR was characterized by the absence of a lag phase, attesting that the microbial population did not need an adjustment time (Figure 2 and Table 2). It is interesting to highlight that although CTR, PM, and 2PM have the same initial and final values (*p* > 0.05), Figure 2 shows a different microbial evolution as confirmed by different LM counts during the sampling time (Table 1), and by DMfit output parameters (λ and µmax, Table 2). No differences were found in µmax and final value for PM and 2PM curves, while differences (*p* < 0.001) were registered in lag phases (Table 2), suggesting that these PE concentrations exert a modulation effect on the LM growth in pork frankfurters. The absence of the lag phase in the CTR and the highest value of µmax compared with the treated groups suggest that phenolic compounds strongly influence LM concentration in the first part of the storage, most likely creating an initial hurdle to microbial growth. Concerning the 4PM group, the analysis performed with ComBase allowed for the definition of a lag phase of approximately 99 h, the lowest µmax (0.005 Log CFU/g/h), and final value 4.158 Log CFU/g. These results suggest that the concentration of polyphenols in the 4PM treatment strongly limit LM proliferation, although the tested frankfurter still represents a meat product able to support LM growth throughout the shelf-life. The treatment of the surface of pork frankfurter led to an elongated lag phase and a decrease in the maximum growth rate of LM in treated samples compared to CTR. The results validate the existence of a relationship between the concentration of polyphenols and the effect observed, which varies depending on the dose. This relationship was observed within the PE concentration range of 29.5–118 mg/mL. It is possible that these differences in the duration of λ and μmax found between treatments can be explained by this dose-dependent effect. Bubonja-Sonje et al. [38] tested the effects of total olive oil polyphenols on LM growth and also noted the dose dependency. According to the literature, this finding may indicate that the first adaptations that bacteria make in response to stressors may be changes in the lag time required to develop tolerance [39]. Indeed, the optimization and elongation of the lag phase in treated samples may represent a strategy adopted by the LM population to tolerate environmental stress, which may correspond, in this study, to the polyphenols present in the food matrix [40,41]. *Olea europea* phenolic compounds are notable for their effectiveness in combating pathogenic microorganisms [20]. Recent studies [42] have shown the efficacy of incorporating hydroxytyrosol in a combination of two different plant extracts against LM in dry fermented sausage; the polyphenolic extract used in this study demonstrated superior efficacy compared to other extracts. Theivendran et al. [37] studied the effect of soy protein edible film containing nisin and grape seed extracts against LM during storage in turkey frankfurter. The authors found that a combination of nisin and natural extract is less effective in a food system than a broth medium. Due to the nutrient-rich environment and solid matrix provided by food products, the efficacy of antimicrobials in the foodstuff is restricted when compared to in vitro studies. In their study, Gadang et al. [16] discovered that an increased acidic environment obtained with the addition of malic acid is essential to improve the inhibitory effects of nisin and grape seed extract against LM in turkey frankfurter. Different surface treatments, active coating, or films can be effective against LM on commercially manufactured vacuum-packaged frankfurters during storage. These treatments include using bacteriocins produced by meat-borne bacteriocinogenic lactobacilli, nisin, organic acids, and natural plant extracts, either alone or in combination [13,16]. However, the majority of these methods lead to changes in the acidity of the product, which negatively impacts sensory attributes such as color, flavor, aroma, and texture [13,16,43]. Regarding the compliance of this product (frankfurter) to the legislation in force, EU Regulation 2073/2005 [44] in Europe has set the safety criteria of 2 Log CFU/g for LM in ready-to-eat products throughout their shelf life. Other countries (e.g., USA), have implemented far more rigorous regulations, establishing a zero-tolerance approach for LM [45]. To meet these requirements, numerous researchers have examined innovative post-processing interventions other than thermal treatment. However, these studies reported different levels of effectiveness of these interventions compared to the untreated and, in some cases, highlighted undesirable alterations of the quality and sensory traits of the products [11,17,37]. In the present study, although the inoculum concentration was equivalent to the threshold limit set by European legislation (2 Log CFU/g) representing, therefore, the worst-case scenario, the 4PM treatment led to a significant limitation in LM growth. Furthermore, in contrast to those previously discussed, the PE treatments utilized in this study did not have any impact on pH or a_w_, as no significant differences for either parameter were observed among the groups. As expected, a_w_ decreased gradually during storage, with the same pattern observed among all treatments (Table 3).

The L* (lightness), a* (intensity of red color), and b* (intensity of yellow color) values of the frankfurters immediately after production (day 0) and at the end of storage (day 28) are shown in Table 4.

Within the same storage time (both T0 and T28), no significant differences (*p >* 0.05) in L*, a*, and b* values were detected between CTR and the treated groups (PM, 2PM, 4PM), corroborating the hypothesis that PE treatment does not affect surface color in frankfurters. Furthermore, it is worth noting that the L*, a*, and b* values found in this study are within the expected range for this type of meat product [46], demonstrating that the treatment did not compromise the visual quality of frankfurters. Table 4 report the ΔE values, representing the overall color difference within each treatment over the days of storage (28 days), compared to the difference of the initial day (day 0).

In all treatments, ΔE values were below 2, even at the end of storage, besides slight recorded differences. ΔE values < 2 are generally considered imperceptible or difficult to perceive by most consumers [47]. Thus, it appears from the study’s findings that color stability remained unchanged even after the longest storage period, in spite of the most concentrated polyphenol treatments. Similar results were obtained by Wang et al. [48], who demonstrate that hops beta acids post-lethality surface treatments applied on meat products had no negative impact on surface color. On the contrary, Gedikoğlu [49] demonstrates that the use of pectin edible coatings prepared with essential oil for inhibiting the growth of LM significantly affects the color of treated meat products. Furthermore, Xi et al. [43] showed that the addition of cranberry powder over 1% in the formulation of frankfurter resulted in significant product pH decline and negatively impacted the color, texture, and sensory attributes of the product. The influence of polyphenol treatment on the color of frankfurters was negligible for the targeted product; however, different kinds of frankfurters (such as white frankfurters) might encounter different impacts from polyphenol treatment, as these compounds are known to alter the color of pale food items [15].

The feasibility of using these olive-derived polyphenols as a treatment for superficial antilisterial effect was examined by sensory analysis. For practical effectiveness, treatments on frankfurters must not alter the product’s sensory qualities; hence, assessments by an untrained sensory panel were carried out to perceive any discernible sensory variation in the treatments.

The results of sensory tests of frankfurters are shown in Table 5, and there was no significant difference among samples at a significance level of α = 0.05 and β = 0.05. The polyphenolic treatment did not change sensory perception even when comparing the highest concentration applied (4PM) with the CTR group.

The results of the preference ranking test are reported in Table 6. The significance of the difference among the samples in each storage day was established through Friedman’s test, comparing the result obtained with a value of the chi-square parameter (χ^2^), calculated as a function of the number of samples and the number of consumers. The value obtained in both determinations (χ^2^ =1.3 for 1 day and χ^2^ =2 for 28 days) was smaller than the critical value at the selected level of significance α = 0.05 and β = 0.05 (χ^2^ = 7.81). Since the calculated χ^2^ is smaller than the tabulated χ^2^ in both assessments, it can be concluded that there are no statistically significant differences among the samples in the two evaluations (ISO 8587:2006) [28]. Additionally, comparison at the beginning and end of storage indicated no significant difference among samples (*p* > 0.05).

Several studies regarding surface antimicrobial treatment resulted in perceived differences between treated and reference samples [50,51], with the treated samples being less preferred by consumers due to the modifications in physicochemical characteristics, in organoleptic properties that, in some cases, led to the presence of off-flavors [13,43,51]. Olive oil polyphenols, as well as other natural compounds, have a chance of being used as natural antimicrobials in meat-based products; thus, they should not negatively influence sensory properties with surface treatments even at high concentrations, although these aspects are strongly related to the extracts’ chemical composition. Sensory characteristics are important attributes for consumers and affect the experienced quality, which ultimately influences the consumer intention to purchase the same product in a posterior moment [51,52].

## 4. Conclusions

Frankfurter surface treatment with olive mill wastewater polyphenolic extract at the concentration tested in this study, especially the highest, effectively hinders the growth of *Listeria monocytogenes*, without causing any changes in the quality of the product or negatively influencing sensory properties. The use of this polyphenolic extract is proposed as an effective obstacle to pathogen growth that, in combination with best hygiene practices of the food business operation, could contain LM proliferation throughout storage of the foodstuff. The definition of the most suitable extract concentration to achieve effective result needs to consider the pathogen contamination level as well as the specific characteristics of the production process and food matrix, also in order to avoid possible sensory depletion of products. Naturally occurring antimicrobials, like olive polyphenols, can be an alternative technology for the long-term preservation, quality control, and safety of meat products—a goal that the food industry is constantly pursuing to meet the needs of contemporary consumers for sustainable, healthy, and rich-in-nutrients meat-based foodstuff.

## 5. Patents

Stymon Natural Products P.C., Patras, Greece (www.stymon.com, accessed on 20 January 2025): patented process (GR1010150, EP4049543A1) and food-grade certificate (certificate released by General Chemical State Laboratory of Greece n. 30/003/000/3810).

## Figures and Tables

**Table 1:** *Listeria monocytogenes* evolution in contaminated frankfurters during 28 days of storage (results are expressed as Log CFU/g).

	Days of Storage					SEM			
	0	7	14	21	28	0.150	S	T	SXT
CTR	2.07 a	5.5 bC	7.95 cD	8.21 cC	8.39 cC		<0.001	<0.001	<0.001
PM	2.00 a	3.43 bB	5.43 cC	7.07 dB	7.74 eB				
2PM	2.15 a	3.10 bB	4.91 cB	6.77 dB	7.66 eB				
4PM	1.94 a	2.31 bA	3.09 cA	3.62 cdA	4.01 dA				

CTR = control group; PM = frankfurter treated with 29.5 mg/mL of total polyphenols; 2PM = frankfurter treated with 59 mg/mL of total polyphenols; 4PM = frankfurter treated with 118 mg/mL of total polyphenols; S = sample; T = time of storage. Different letters in the same row (a, b, c, d, e) indicate differences among mean values of the same treatment in different time period (*p* < 0.001). Different letters in the same column (A, B, C, D) indicate differences among mean values of the treatment (*p* < 0.001); SEM = standard error of the means.

**Table 2:** Growth kinetics parameters estimated by the DMFit program for *Listeria monocytogenes* population in the four treatments of pork frankfurters.

	CTR	PM	2PM	4PM	SEM	*p*-Value
Initial value(Log CFU/g)	2.085	1.997	2.152	1.940	0.047	0.177
λ (h)	n.d.	52.059 a	97.079 b	99.741 b	15.469	<0.001
µmax (Log CFU/g/h)	0.020 c	0.012 b	0.012 b	0.005 a	0.003	<0.001
Final value(Log CFU/g)	8.268 b	7.763 b	7.764 b	4.158 a	0.951	<0.001

λ = lag phase (h); µmax = maximum growth rate (Log/CFU/g/h); final value (Log/CFU/g); CTR = control group; PM = frankfurter treated with 29.5 mg/mL of total polyphenols; 2PM = frankfurter treated with 59 mg/mL of total polyphenols; 4PM = frankfurter treated with 118 mg/mL of total polyphenols. Different letters in the same row (a, b, c) indicate differences among mean values of the treatments (*p* < 0.001); SEM = standard error of the means; n.d. = not detected.

**Table 3:** Physicochemical characteristics of pork frankfurter for the four treatments during storage.

		Days of Storage					SEM			
	Sample	0	7	14	21	28		T	S	TxS
pH	CTR	6.09 b	6.00 ab	5.97 ab	5.94 ab	5.90 a	0.038	0.006	0.323	0.925
	PM	5.97	5.97	5.95	5.93	5.91				
	2PM	6.05	6.00	5.98	5.96	5.93				
	4PM	5.98	5.97	5.95	5.95	5.93				
a*_w_*	CTR	0.978 c	0.975 bc	0.973 b	0.972 ab	0.968 a	0.001	<0.01	0.042	0.554
	PM	0.978 b	0.976 b	0.974 ab	0.972 a	0.971 a				
	2PM	0.976 b	0.974 b	0.973 b	0.973 b	0.967 a				
	4PM	0.979 c	0.975 bc	0.974 b	0.973 ab	0.970 a				

CTR = control group; PM = frankfurter treated with 29.5 mg/mL of total polyphenols; 2PM = frankfurter treated with 59 mg/mL of total polyphenols; 4PM = frankfurter treated with 118 mg/mL of total polyphenols. S = sample; T = time of treatment. Different letters in the same row (a, b, c) indicate differences among values of the same treatment in different time period (*p* < 0.001); SEM = standard error of the means.

**Table 4:** L*, a*, and b* values and global color difference (ΔE) within each treatment of the pork frankfurter after 0 day and 28 days of storage.

	Days of Storage	Samples				SEM	T	S	TxS
		CTR	PM	2PM	4PM				
L*	0	55.82	55.27	55.18	54.76	0.417	0.112	0.850	0.337
	28	54.50	54.97	54.59	54.99				
a*	0	15.30 B	15.09	15.02	14.91	0.184	0.001	0.480	0.099
	28	14.20 A	14.91	14.63	14.57				
b*	0	11.87	12.18	12.44	12.79	0.447	0.479	0.858	0.600
	28	12.54	12.75	12.55	12.36				
						SEM	*p*-value		
ΔE		1.99 b	1.62 ab	1.89 b	1.23 a	0.170	0.016		

CTR = control group; PM = frankfurter treated with 29.5 mg/mL of total polyphenols; 2PM = frankfurter treated with 59 mg/mL of total polyphenols; 4PM = frankfurter treated with 118 mg/mL of total polyphenols. S = sample; T = time of treatment. Different letters in the same row (a, b) indicate differences between mean values for different experimental groups (*p* < 0.001). Different letters in the same column (A, B) indicate differences among mean values of the same treatment in different time period (*p* < 0.001). Different letters in the same raw (a, b) indicate differences among mean values of the treatments. SEM = standard error of the means. ΔE = [(L* − L*_0_)^2^ + (a* − a*_0_)^2^ + (b* − b*_0_)^2^]^0.5^.

**Table 5:** Results from triangle tests with frankfurter-type sausage.

Session	Compared Samples	Number of Untrained Assessors	Correctly Identified	Correctly Identified %	Significance Differences (α = 0.05; β = 0.05)
1	CTR vs. PM	30	5	16.7	no
2	CTR vs. 2PM	30	8	26.7	no
3	CTR vs. 4PM	30	11	36.7	no
4	PM vs. 2PM	30	4	13.3	no
5	PM vs. 4PM	30	5	16.7	no
6	2PM vs. 4PM	30	7	23.3	no

CTR = control group; PM = frankfurter treated with 29.5 mg/mL of total polyphenols; 2PM = frankfurter treated with 59 mg/mL of total polyphenols; 4PM = frankfurter treated with 118 mg/mL of total polyphenols. In accordance with ISO 4120:2007 [27], a minimum of 15/30 correct answers is required to determine that there is a discernible difference (α = 0.05; β = 0.05) among samples.

**Table 6:** Sum of orders obtained in the preference ranking test.

	Rank Sum				*χ* ^2^	*p*-Value
	CTR	PM	2PM	4PM		
1 day of storage	33	31	26	30	1.3	0.729
28 days of storage	28	26	34	32	2	0.572

CTR = control group; PM = frankfurter treated with 29.5 mg/mL of total polyphenols; 2PM = frankfurter treated with 59 mg/mL of total polyphenols; 4PM = frankfurter treated with 118 mg/mL of total polyphenols. The significance of the difference among the samples in each storage day was established through Friedman’s test, ISO 8587:2006 [28], the critical value of χ^2^ was tabulated for 12 assessors; 4 samples = 7.81. Differences among samples are significant if χ^2^ > 7.81.

## Data Availability

The original contributions presented in the study are included in the article, further inquiries can be directed to the corresponding author.
